# Genome-Wide RNAi Screen Identifies Novel Host Proteins Required for Alphavirus Entry

**DOI:** 10.1371/journal.ppat.1003835

**Published:** 2013-12-19

**Authors:** Yaw Shin Ooi, Katie M. Stiles, Catherine Y. Liu, Gwen M. Taylor, Margaret Kielian

**Affiliations:** Department of Cell Biology, Albert Einstein College of Medicine, Bronx, New York, New York, United States of America; Vanderbilt University School of Medicine, United States of America

## Abstract

The enveloped alphaviruses include important and emerging human pathogens such as Chikungunya virus and Eastern equine encephalitis virus. Alphaviruses enter cells by clathrin-mediated endocytosis, and exit by budding from the plasma membrane. While there has been considerable progress in defining the structure and function of the viral proteins, relatively little is known about the host factors involved in alphavirus infection. We used a genome-wide siRNA screen to identify host factors that promote or inhibit alphavirus infection in human cells. Fuzzy homologue (FUZ), a protein with reported roles in planar cell polarity and cilia biogenesis, was required for the clathrin-dependent internalization of both alphaviruses and the classical endocytic ligand transferrin. The tetraspanin membrane protein TSPAN9 was critical for the efficient fusion of low pH-triggered virus with the endosome membrane. FUZ and TSPAN9 were broadly required for infection by the alphaviruses Sindbis virus, Semliki Forest virus, and Chikungunya virus, but were not required by the structurally-related flavivirus Dengue virus. Our results highlight the unanticipated functions of FUZ and TSPAN9 in distinct steps of alphavirus entry and suggest novel host proteins that may serve as targets for antiviral therapy.

## Author Summary

Alphaviruses are a group of small enveloped viruses that include important human pathogens for which there are no antiviral therapies or vaccines. Alphaviruses enter host cells by receptor-mediated endocytosis and low pH-triggered membrane fusion, and exit by budding from the host cell plasma membrane. The roles of host cell proteins in these events are not well understood in spite of extensive studies. Here we performed a screen using small interfering RNAs to identify host factors involved in alphavirus infection of human cells. We defined the mechanism of two novel host proteins that promote alphavirus entry. Fuzzy homologue (FUZ), a protein with roles in cilia biogenesis, promoted endocytosis of both alphaviruses and a well-studied endocytic cargo, transferrin. The tetraspanin membrane protein, TSPAN9, did not significantly affect endocytic uptake or acidification, but was critical for the efficient fusion of the virus in the endosome. These two proteins were required for infection by several different alphaviruses, suggesting that they may be useful targets for drugs to prevent alphavirus infection.

## Introduction

Alphaviruses are small enveloped plus-sense RNA viruses that are transmitted in nature by mosquito vectors [1]. These viruses can cause debilitating illness, severe arthritis, and encephalitis in humans, as in the case of the recently emerged alphavirus Chikungunya virus and the encephalitic alphaviruses Eastern, Western, and Venezuelan equine encephalitis virus [2], [3].

Alphaviruses have highly organized structures, with an internal nucleocapsid core surrounded by a viral membrane containing a lattice of the E2 and E1 proteins [4], [5]. They infect cells via binding to receptors on the plasma membrane and internalization by clathrin-mediated endocytosis [1], [6], [7]. The E1 envelope protein then mediates low pH-dependent fusion of the virus and endosome membranes, releasing the nucleocapsid into the cytoplasm. Virus replication occurs in the cytoplasm and the envelope proteins are transported via the host secretory pathway to the cell surface where new virus particles bud [1].

While there has been considerable progress in defining the structure and function of the viral proteins, relatively little is known about the host factors involved in alphavirus infection. Here we used a genome-wide siRNA screen to identify such host factors in human cells, and defined the mechanisms of several novel host proteins that promote alphavirus infection. These factors were involved in distinct steps of the virus entry pathway: the protein FUZ promoted endocytic uptake and the protein TSPAN9 promoted fusion of internalized, low pH-triggered virus in the endosome.

## Results

### siRNA screen

**Figure 1:**
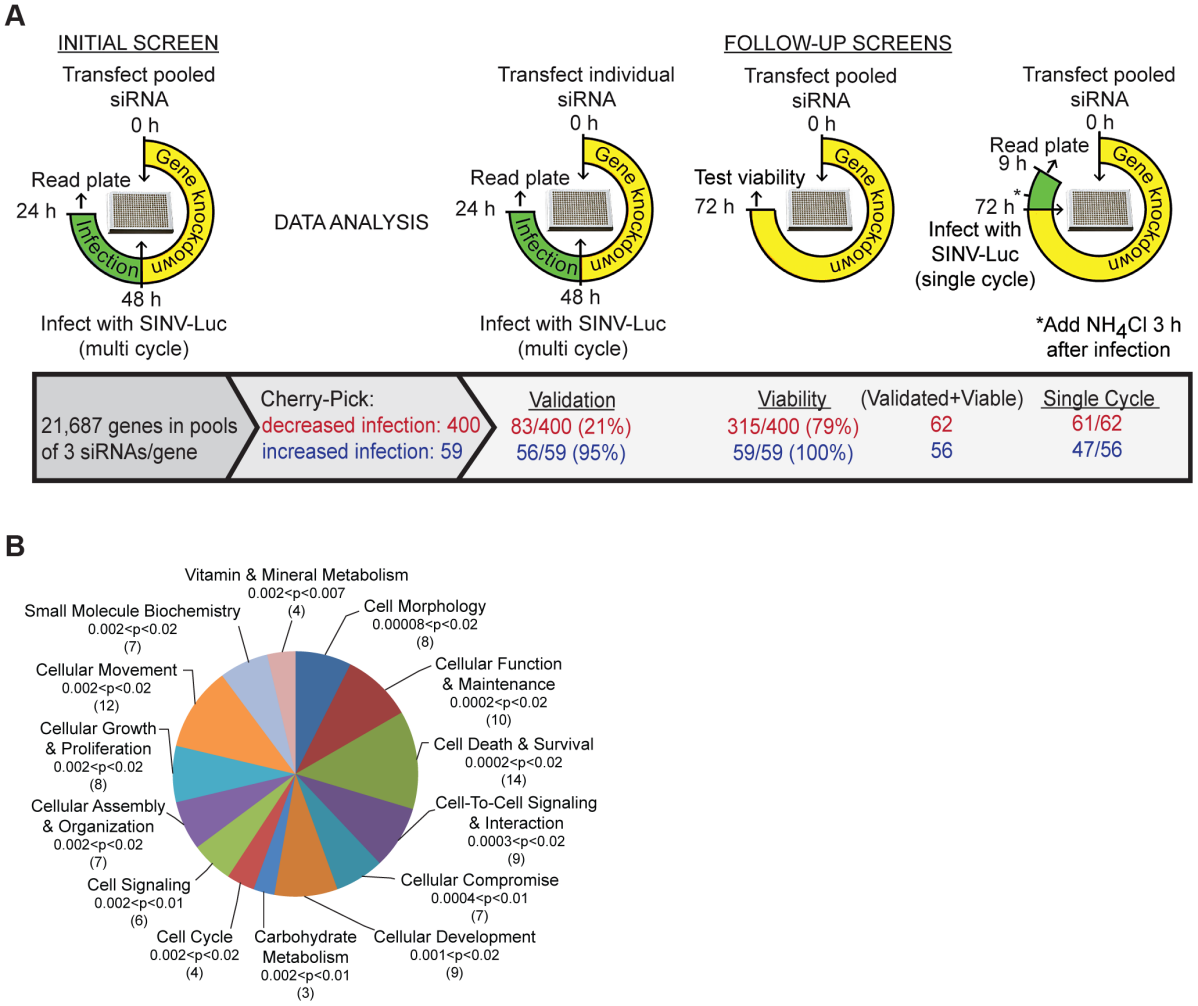
Sindbis virus genome-wide RNAi screen. A. Strategy for initial and follow-up screens of Sindbis host factors. Initial screen was performed using pooled siRNAs and SINV infection at low multiplicity for 24 h. Selected siRNAs were tested individually under the same conditions; validation refers to those host factors in which ≥2/3 siRNAs inhibited or promoted SINV infection. Pooled siRNAs were also tested for effects on cell viability and for inhibition of single-cycle SINV infection. B. Ingenuity Pathway analysis of 55 proteins identified by the screen as promoting SINV-Luc infection. The top 14 overrepresented categories of molecular and cellular functions are shown, with the significance (p-values) and number of proteins indicated for each category in parentheses.

To identify novel host proteins involved in various aspects of alphavirus infection, we performed a genome-wide RNA interference screen using the prototype alphavirus Sindbis virus, engineered to express firefly luciferase in the cytoplasm of infected cells (SINV-Luc). Conditions were optimized to detect multiple cycles of virus infection, which were dependent on both clathrin and endosomal acidification (Fig. S1A, B). A total of 21,687 genes were targeted in human U-2 OS cells using pools of 3 siRNAs per gene, and screened for effects on SINV-Luc infection (Figs. 1A and S1C, D) (Table S1).

Analysis of robust z-scores and comparison with the positive and negative controls on each plate were used to identify 400 genes that may promote alphavirus infection and 59 genes that potentially encode antiviral factors (Materials and Methods, Fig. 1A). The siRNAs targeting these 459 genes were then tested to confirm effects on multi-cycle infection, to determine effects on single cycle infection (Fig. S1E), and to eliminate cytotoxic siRNA pools. 356 of the 400 siRNA pools that inhibited virus infection showed the same phenotype upon retesting, and 83 reproduced this effect with multiple (2–3) siRNAs. All of the 59 siRNA pools that promoted virus infection were positive upon retesting, and 56 out of 59 showed this phenotype with multiple siRNAs. After the elimination of siRNA pools in which only 1 siRNA reproduced the original phenotype and pools that decreased cell viability, we identified 118 SINV host factors: 62 factors that promote SINV infection (Table S2), and 56 factors that inhibit SINV infection (Table S3). Comparison of multi-cycle and single cycle infection assays suggested that the majority of these host factors are involved in initial SINV entry and replication (Fig. 1A, last column, and Tables S2 and S3). Comparison with screens for proteins involved in transferrin and epidermal growth factor entry [8] and clathrin-coated vesicle function [9] showed that ∼37% of our 118 SINV host factors are implicated in some aspect of endocytosis (Table S4). About 20% of our SINV host factors were identified in screens for host genes involved in infection by other animal viruses (Table S5).

The 62 host factors that promote SINV infection were grouped into functions with p values indicating the significance of their over-representation vs. the original siRNA library (Fig. 1B). They include host proteins known to be involved in alphavirus endocytic entry, such as vacuolar ATPase subunit ATP6V0C and dynamin 2, and NDST1, which is involved in sulfated proteoglycan synthesis and would thus promote SINV-Luc attachment to host cells [6], [10]. All three of these proteins were also identified in endocytosis screens and tests of other viruses (Tables S4 and S5). Our initial screen also ranked vATPase subunits ATP6AP2, ATP6V1B1 and ATP60E within the top 1000 proviral hits (Table S1). The 56 host factors that inhibit SINV infection include MAP1LC3B, which is involved in cargo recognition during selective autophagy, a pathway that protects against SINV infection [11]. Our screen did not identify NRAMP2, the iron transporter that acts as a receptor for SINV [7], as a host factor, perhaps due to its genetic redundancy in mammalian cells [7] or to differences in the libraries, host cells, etc. However, the iron-sensing protein FBXL5 was identified as an inhibitor of SINV infection (Table S3). FBXL5 promotes the degradation of the iron regulatory proteins that stabilize the mRNA for NRAMP2 [12]. Thus, our screen identified a number of factors with clearly identifiable functions in either promoting or inhibiting SINV infection.

### Defining inhibition by ARCN1, FUZ and TSPAN9 depletion

We chose three host factors that promote SINV infection for further study, based on the strength of their phenotypes and their possible novel roles in alphavirus infection. Archain1 (ARCN1) is the delta subunit of the COPI coatomer complex, which mediates vesicular traffic in both the secretory and endocytic pathways [13] (Table S4). COPI proteins can play roles in the internalization, RNA replication, and morphogenesis of a variety of viruses [14]–[16] (Table S5), but their role in alphavirus infection has not been defined [17]. Our initial screen also ranked the COPI coatomer proteins COPA, COPB, COPB2 and COPG within the top 1000 proviral hits (Table S1). Fuzzy homologue (FUZ) is an effector protein involved in planar cell polarity, ciliogenesis, and mammalian embryonic development [18]–[21]. FUZ has no reported roles in virus infection or endocytosis. TSPAN9 [22] is a little characterized member of the tetraspanin family of membrane proteins, which have a wide variety of reported functions including acting as membrane organizers, modulating effectors and signaling complexes, and affecting cellular adhesion, motility and membrane fusion [23]. TSPAN9 was identified in a broad endocytosis screen (Table S4) but has no known roles in promoting virus infection (Table S5).

**Figure 2:**
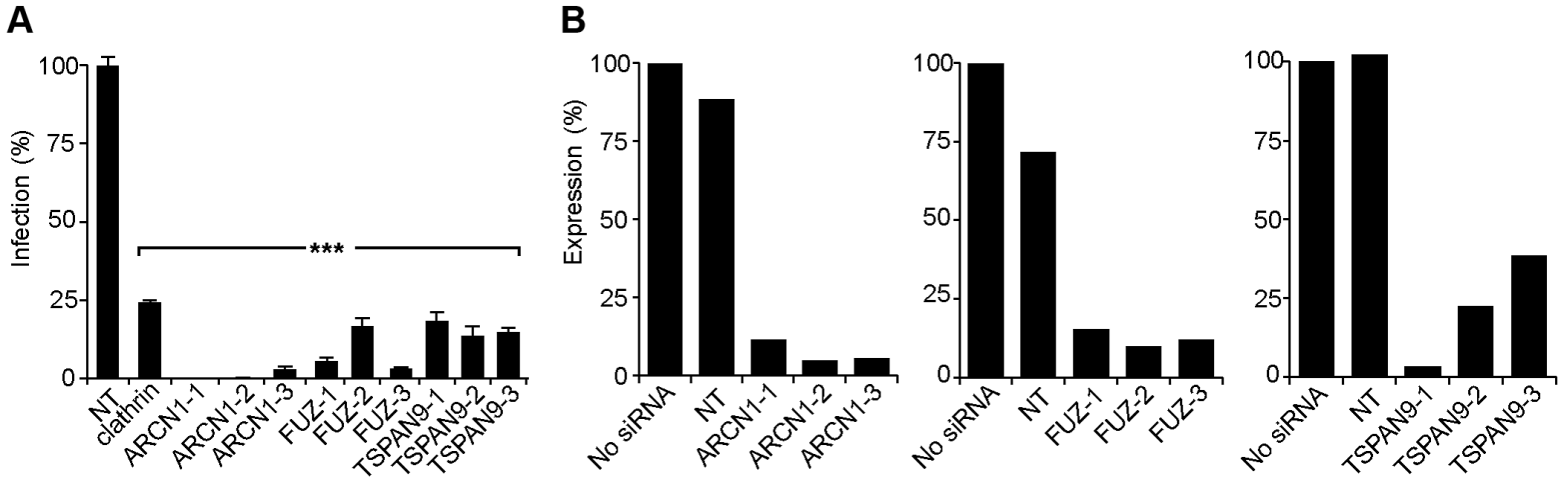
Silencing of ARCN1, FUZ, and TSPAN9 in human U-2 OS cells inhibits alphavirus infection. A. U-2 OS cells were transfected with the indicated single siRNAs. At 48 h post-transfection, cells were infected with SINV-Luc (MOI = 1) and luciferase expression was quantitated 24 h post-infection. Data were normalized to the non-targeting siRNA control (NT). Bar graph represents the mean +/− SEM of 3 determinations (*** indicates p-values<0.001). B. Quantigene assay of mRNA levels in U-2 OS cells 48 h after transfection with the indicated siRNAs, with data normalized to non-transfected control cells.

**Figure 3:**
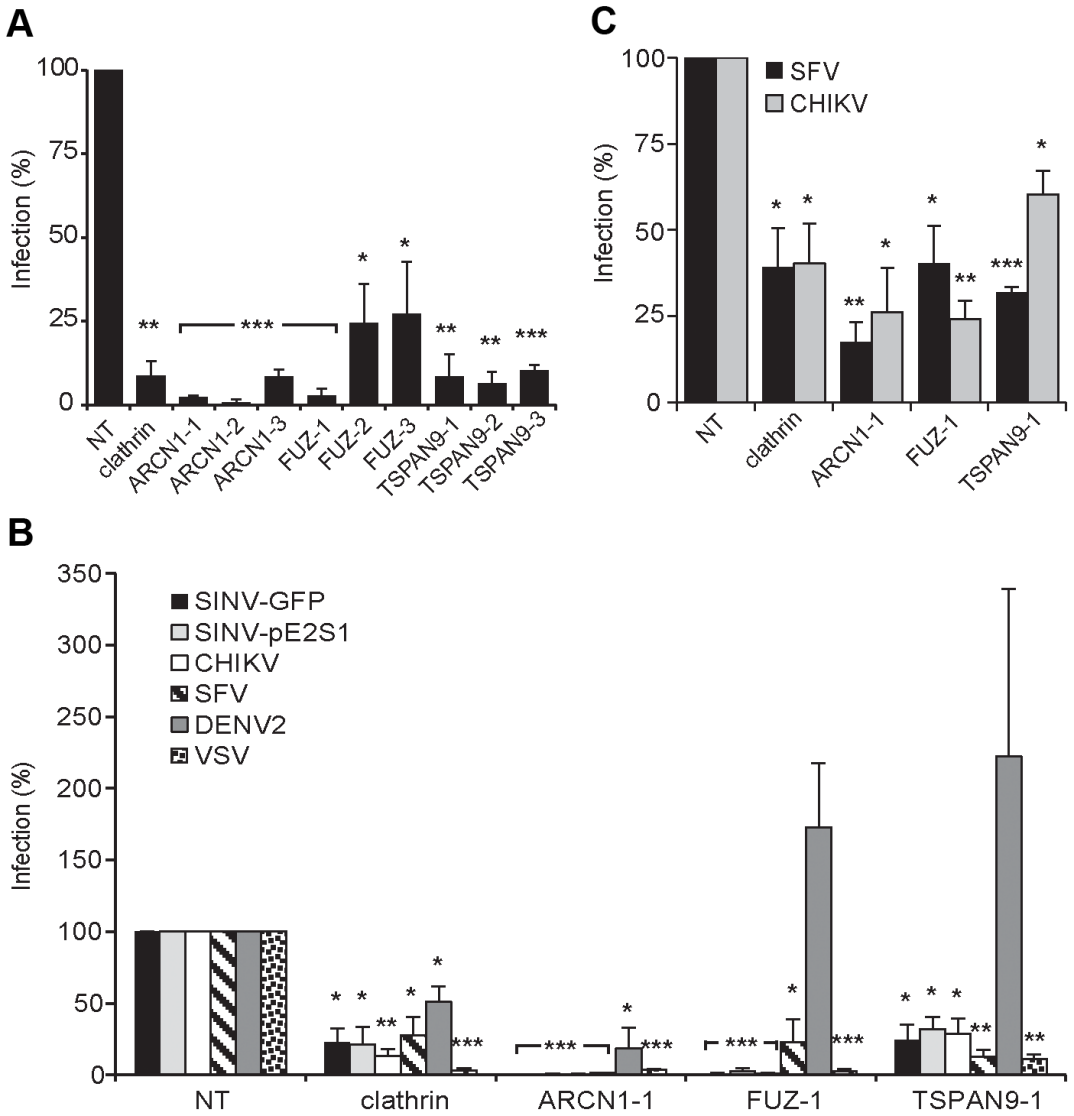
Tests of ARCN1, FUZ, and TSPAN9 with other host cells and viruses. A. HeLa cells were transfected as in (Fig. 2A), infected with SINV-GFP (MOI = 50) and infection quantitated at 24 h post-infection by fluorescence microscopy. B. U-2 OS cells were transfected as in Fig. 2A and infection by the indicated viruses was tested as detailed in the methods. C. Primary human umbilical vein endothelial cells were transfected with the indicated siRNAs, incubated for 60 h, infected with SFV or CHIKV, and infection quantitated at 8–12 h post-infection by fluorescence microscopy. Values in A–C were normalized to a non-targeting siRNA control (NT), and the data in each panel represent the mean +/− SEM of 3 independent experiments (*p<0.05, **p<0.01, ***p<0.001).

For each of these factors, we confirmed that transfection of the 3 individual siRNAs into U-2 OS cells resulted in inhibition of SINV-Luc infection (Fig. 2A). Quantigene analysis of U-2 OS cells demonstrated that target gene mRNA levels were reduced to <15% of the control by all 3 ARCN1 and FUZ siRNAs and for TSPAN9-1 siRNA, while TSPAN9-2 and -3 siRNAs caused reductions to 22–38 % of control (Fig. 2B). siRNA transfection of HeLa cells produced similar inhibition of infection (Fig. 3A), confirming that the effect is not cell-type specific. We also confirmed both inhibition of virus infection and decreased mRNA levels using either an additional shRNA or an esiRNA (Fig. S2). We selected the individual siRNAs ARCN1-1, FUZ-1, and TSPAN9-1 for further studies. Infection by the alphaviruses SINV-GFP, SINV-pE2S1, Semliki Forest virus (SFV), and Chikungunya virus (CHIKV) was inhibited by these siRNAs to a level similar to the inhibition observed for SINV-Luc (Fig. 3B). SINV-pE2S1 is a strain of SINV whose infection and attachment are independent of heparan sulfate proteoglycans [10]. Thus, the requirement for ARCN1, FUZ, and TSPAN9 is conserved among several different alphaviruses. Infection by the rhabdovirus vesicular stomatitis virus (VSV) was similarly inhibited. In contrast, while infection by the flavivirus Dengue virus 2 (DENV2) was inhibited by clathrin or ARCN1 depletion (Fig. 3B) and was dependent on endosomal acidification (data not shown), it was resistant to depletion of either FUZ or TSPAN9 (Fig. 3B). Human umbilical vein endothelial cells (HUVEC) were transfected with the indicated siRNAs and tested for infection with SFV and CHIKV (Fig. 3C). Depletion of clathrin, ARCN1, FUZ or TSPAN9 significantly inhibited infection by both viruses, thus demonstrating a requirement for these proteins during alphavirus infection of primary human cells.

**Figure 4:**
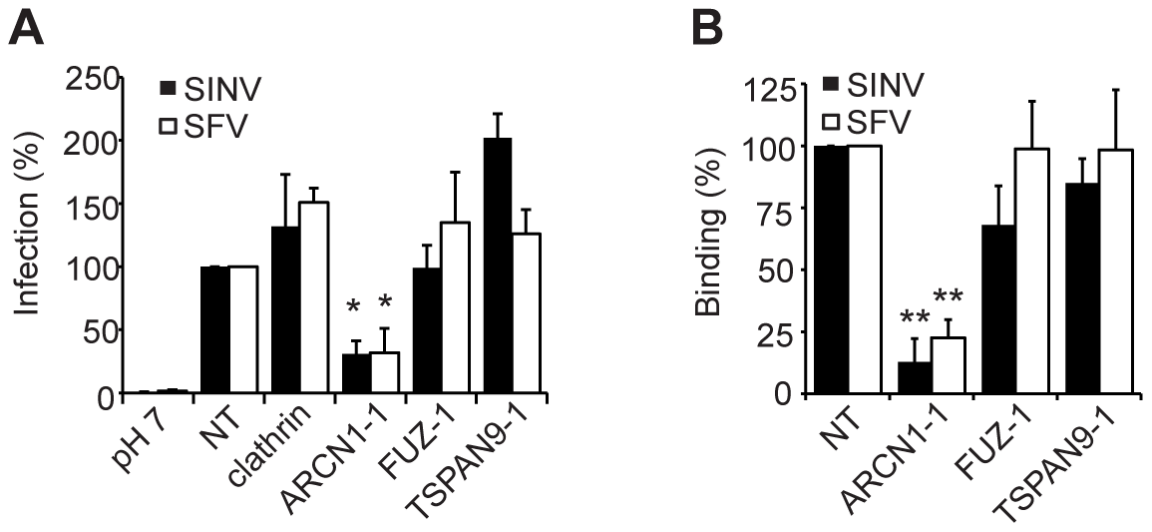
ARCN1 depletion inhibits alphavirus binding to host cells. U-2 OS cells were transfected as in Fig. 2A and tested as follows: A. Virus fusion-infection. SINV or SFV was pre-bound to cells on ice and treated at low pH to trigger virus fusion with the plasma membrane. Infected cells were quantitated by immunofluorescence. B. Cell surface binding. SINV or SFV was bound to cells on ice and detected by immunofluorescence. A–B each represent the mean +/− SEM of 3–4 independent experiments with data normalized to the NT control (*p<0.05, **p<0.01).

Depletion of ARCN1, FUZ or TSPAN9 inhibited single cycle SINV infection (Fig. 1A and Table S2). We therefore analyzed the initial stage(s) of the alphavirus lifecycle that might be dependent on these proteins. Fusion of prebound SINV-GFP or SFV with the plasma membrane bypassed the requirement for clathrin, FUZ and TSPAN9 in infection (Fig. 4A), suggesting that like clathrin, FUZ and TSPAN9 are involved in virus entry steps. In contrast, the requirement for ARCN1 was not bypassed (Fig. 4A). Direct binding experiments showed a decrease of >80% in SINV or SFV binding to ARCN1-depleted cells but no decrease with FUZ or TSPAN9-depleted cells (Figs. 4B and S3A). Viral RNA transfection experiments showed a moderate but statistically significant decrease in SINV and SFV infection of ARCN1-depleted cells, most likely through effects on viral RNA transcription/replication (Fig. S3B, C). Thus, ARCN1 depletion most significantly affected alphavirus infection by a strong inhibition of cell surface binding, presumably due to reduced receptor delivery to the plasma membrane. Inhibition was observed for both SINV, which uses NRAMP2 as a receptor [7], and SFV, for which the receptor has not been identified. Decreased virus-cell binding is a mechanism of inhibition that has not been previously described in other studies of the role of the COPI complex in virus infection. While COPI depletion has been reported to inhibit endocytic uptake and traffic [15], [17], its potential effects on alphavirus endocytosis would not be detectable in our system due to the upstream block in alphavirus attachment.

### FUZ depletion inhibits virus uptake

**Figure 5:**
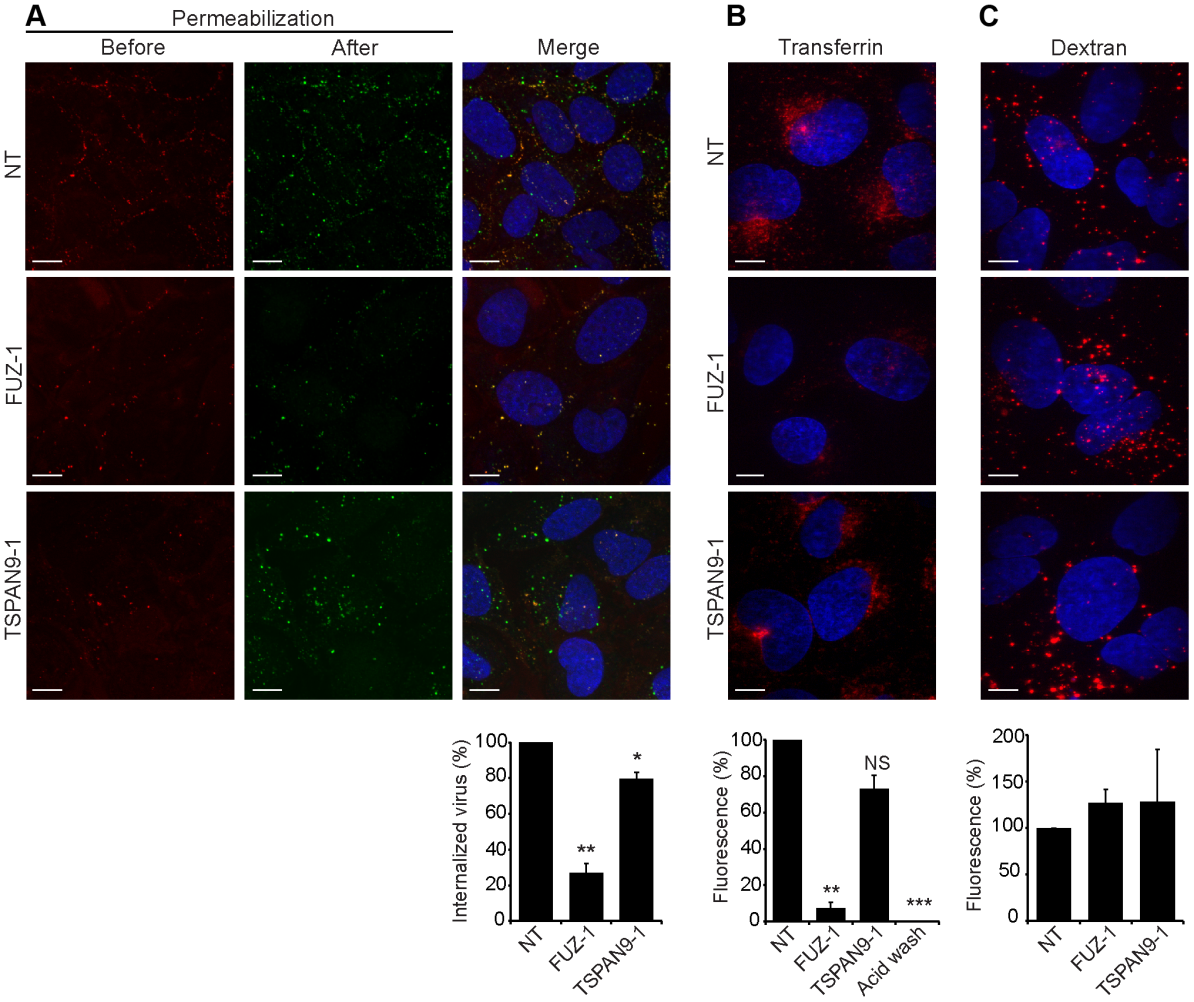
FUZ depletion inhibits alphavirus endocytosis. U-2 OS cells were transfected as in Fig. 2 A and tested as follows: A. SFV endocytosis. Cells with pre-bound SFV were incubated at 37°C for 30 min to permit endocytosis, and then fixed and stained with rabbit antibody to the envelope proteins before and after cell permeabilization, conditions that detect virus remaining on the cell surface (red/yellow) vs. internalized virus (green), respectively. B. Transferrin uptake. Cells were pre-bound with fluorescent transferrin on ice, incubated for 30 min at 37°C to permit endocytosis, and washed at pH 2.5 to strip off non-internalized transferrin before fixation. The control was acid-washed prior to 37°C incubation. C. Fluid phase uptake. Cells were imaged after incubation with fluorescent dextran for 3 h at 37°C followed by a 2 h chase. Panels A–C show confocal extended focus images (bar = 10 µM). Bar graphs with each panel represent the mean +/− SEM of 3 independent experiments with data normalized to NT control (*p<0.05, **p<0.01, ***p<0.001). All three siRNAs to FUZ and TSPAN9 produced similar results (data not shown).

To define the roles of FUZ and TSPAN9 in the virus entry pathway, we took advantage of our specific reagents for the steps in SFV entry. We first tested whether depletion of either protein inhibited SFV endocytosis. FUZ-depleted cells showed a striking reduction in SFV internalization, to ∼20% of the level in control cells (Fig. 5A). TSPAN9-depleted cells showed a small decrease in SFV internalization, to ∼80% of control cells (Fig. 5A). We then tested the uptake of transferrin, a ligand that is internalized by clathrin-mediated endocytosis, accumulated in early endosome compartments, and recycled to the plasma membrane [24]. Labeling of FUZ-depleted cells by internalized transferrin was reduced to ∼10% of the level in control cells, while labeling of TSPAN9-depleted cells showed a modest decrease that was not statistically significant (Fig. 5B). Uptake of LDL, which is also internalized by clathrin-mediated endocytosis, was also decreased in the FUZ-depleted cells (Fig. S4). In contrast, the fluid-phase endocytic marker fluorescent dextran produced equivalent labeling of control, TSPAN9-depleted and FUZ-depleted cells (Fig. 5C). Lysotracker staining indicated that general endosome/lysosome acidification was also unimpaired by FUZ or TSPAN9 depletion (data not shown).

### TSPAN9 depletion inhibits intracellular virus fusion

**Figure 6:**
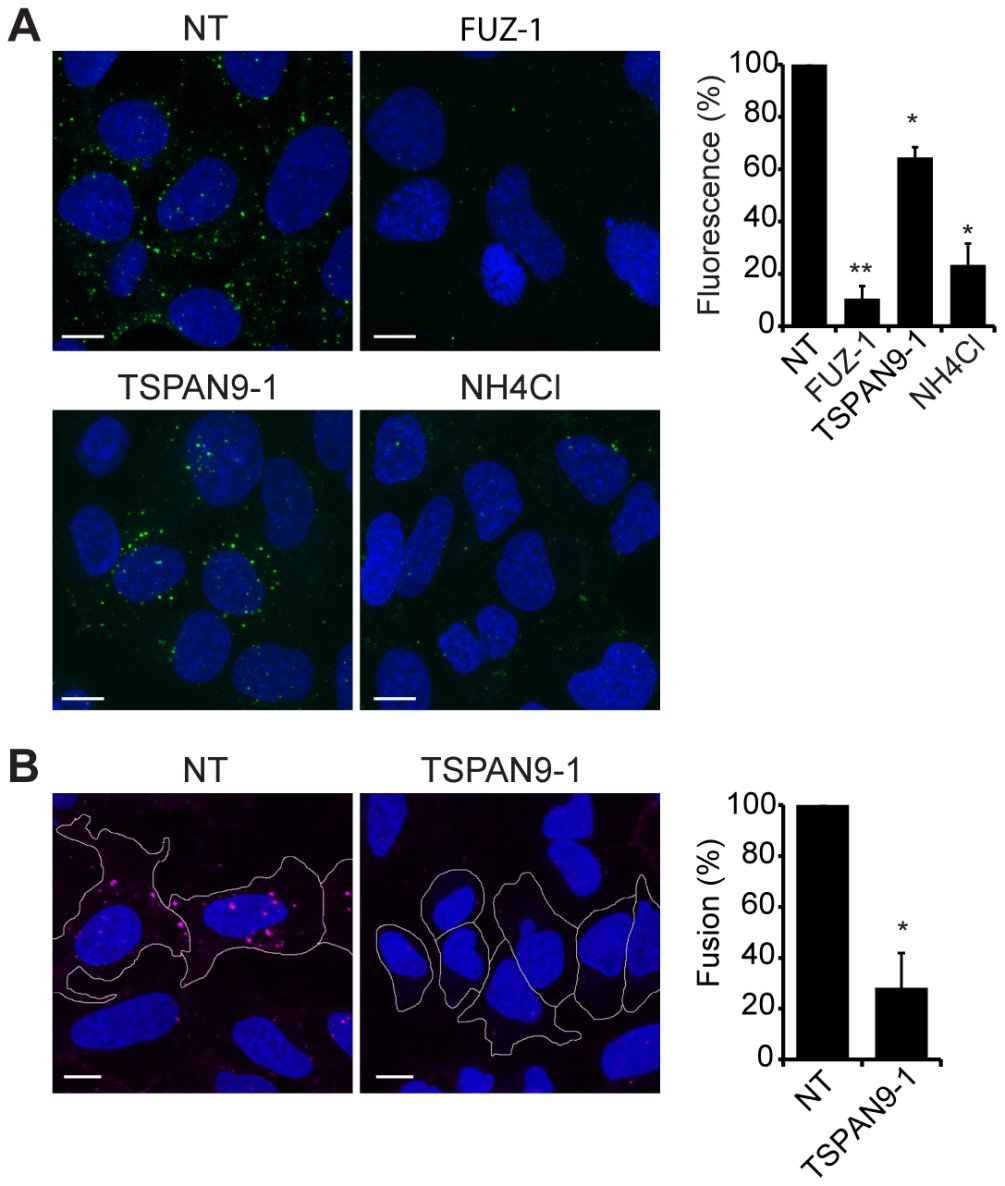
TSPAN9 depletion inhibits alphavirus membrane fusion. U-2 OS cells were transfected as in Fig. 2 A and tested as follows: A. Assay of the low pH-triggered E1 conformational change. Cells with pre-bound SFV were incubated at 37°C for 20 min to permit endocytosis. Cells were fixed and permeabilized, and the low pH conformation of viral E1 was quantitated in cells by staining with mAb E1a-1. Control shows cells in which the 37°C incubation was carried out in medium containing 20 mM NH_4_Cl to block endosome acidification. B. Virus fusion assay. Cells with pre-bound DiD-labeled SFV were incubated at 37°C for 20 min, fixed, and the signal from fused virus particles quantitated by fluorescence microscopy. Cells were then permeabilized, stained with mAb E1a-1, and the signal from the low pH conformation of E1 quantitated. Data from the DiD lipid mixing fusion assay were corrected for the difference in the E1a-1-positive signal in the TSPAN9-depleted cells. Panels A–B show confocal extended focus images (bar = 10 µM). Bar graphs with each panel represent the mean +/− SEM of 3 independent experiments with data normalized to NT control (*p<0.05, **p<0.01).

Upon exposure to the low pH environment of the early endosome, the alphavirus fusion protein E1 undergoes acid-induced conformational changes that can be detected by the conformation-specific SFV monoclonal antibody E1a-1 [25]. Since TSPAN9 depletion did not strongly inhibit SFV uptake, we tested if the depleted cells could trigger the E1 conformational change (Fig. 6A). Control cells showed abundant E1a-1staining, and NH_4_Cl treatment confirmed that staining was dependent on endosomal acidification. Little E1a-1 staining (<10% of control) was observed in FUZ-depleted cells, in keeping with their strong decrease in SFV endocytosis. TSPAN9-depleted cells supported the E1 conformational change, showing a small decrease in E1a-1 staining comparable to the reduction in virus endocytosis. These results suggested that the block in TSPAN9-depleted cells was at a step after the acid-induced conformational change in E1. We directly assayed intracellular virus fusion using SFV labeled with a self-quenching concentration of the lipophilic fluorescent dye DiD, and using E1a-1 staining to correct to equivalent levels of internalized, acid-triggered virus. Membrane fusion of E1a-1-positive SFV was decreased by ∼80% in TSPAN9-depleted cells (Fig. 6B).

## Discussion

Our data demonstrated that ARCN1 depletion blocked alphavirus-cell binding, and identified two novel host factors, FUZ and TSPAN9, that promote alphavirus entry and infection. FUZ is a cytoplasmic protein of ∼46 kDa, and was originally identified in *Drosophila* as Fuzzy, an effector of planar cell polarity [26]. FUZ knockouts in mice cause embryonic lethality and neural tube defects [18], [19], and human FUZ mutations are associated with neural tube defects [27]. While these developmental phenotypes are complex, defects in FUZ act at least in part through their effects on formation of the primary cilium, a structure with roles in signaling pathways [28], [29]. FUZ promotes ciliogenesis, and has been shown to affect the intraflagellar transport system and the localization of the small GTPase rab8 [20], [21]. The relative importance of FUZ in trafficking of internal versus membrane components of the cilium and the potential roles of FUZ in other aspects of membrane traffic remain unclear [29].

Our data indicate that, in addition to its functions in ciliogenesis and development, FUZ plays an important role in clathrin-mediated endocytosis. FUZ depletion blocked alphavirus endocytic uptake and inhibited intracellular transferrin and LDL accumulation. While infection by a panel of alphaviruses and VSV was inhibited by FUZ depletion, both fluid phase uptake and DENV infection were unaffected. This may reflect differences in the entry pathway of DENV versus the pathways of alphaviruses and VSV. All of these viruses are dependent on clathrin, but only DENV fusion occurs in late endosomes [30] and is promoted by acidic phospholipids [31], suggesting a parallel clathrin pathway that is not dependent on FUZ. FUZ mRNA was reported to be expressed in all assayed human tissues [32], suggesting that FUZ could play a role in clathrin-mediated endocytosis in a wide variety of cell types. Although FUZ is essential for mouse development, its depletion by siRNA targeting in primary cells and cell lines did not cause cytotoxic effects within the time scale of our experiments. Given that cells can adapt to the depletion or inhibition of specific endocytic routes by up-regulation of alternative pathways [33], [34], the acute depletion of FUZ will be an important tool to define its mechanism.

TSPAN9 is a 4-pass membrane protein of ∼27 kDa with the N- and C-termini located in the cytoplasm. Its large extracellular loop contains a glycosylation site and 6 cysteine residues, placing it in group 2b of the tetraspanin superfamily [23]. TSPAN9 mRNA is expressed at similar levels in all assayed human tissues [32] and is also expressed in a wide variety of cell lines [22]. Immunolocalization studies in U-2 OS cells stably overexpressing TSPAN9 showed that it is localized at the plasma membrane and on vesicular structures in the cytoplasm (Fig. S5A). TSPAN9 was detected in a systems survey that identified ∼4600 factors involved in transferrin and epidermal growth factor endocytosis [8]. While our data document some reduction in both transferrin and alphavirus endocytosis, this was a relatively minor effect of TSPAN9 depletion. While SFV binding, endocytosis, and low pH-triggered E1 conformational changes occurred quite efficiently in depleted cells, the fusion of internalized virus was strongly blocked. Overexpression of TSPAN9 in U-2 OS cells increased the efficiency of SFV infection compared to that of control cells (Fig. S5B). Together our data support a role for TSPAN9 in a late step of alphavirus entry. TSPAN9 could be required for the correct routing of the virus to the early endosome compartment, or for maintaining the early endosome membrane in a fusion-permissive state. Such models could explain why DENV, which fuses in the late endosome, is resistant to TSPAN9 depletion.

In conclusion, our study reveals distinct and unexpected roles for FUZ and TSPAN9 in alphavirus entry. FUZ and TSPAN9 are conserved from mosquitoes to humans, and thus our results suggest that these proteins play important roles during alphavirus infection of both the mosquito vector and the mammalian host. Defining these roles will be important to understanding the early steps of alphavirus infection and the utility of FUZ and TSPAN9 as novel antiviral targets.

## Materials and Methods

### Cell lines

U-2 OS cells were maintained in McCoy's 5A medium with 10% fetal bovine serum (FBS), 100 U penicillin/ml, 100 µg streptomycin/ml, and L-glutamine at 37°C. HeLa cells were maintained in DMEM with 10% FBS, 100 U penicillin/ml, 100 µg streptomycin/ml, and L-glutamine at 37°C. To make cell stocks for transfection, U-2 OS or HeLa cells were split 4 days prior to siRNA transfection and seeded at 1×10^6^ in 75 cm^2^ flasks.

### Sindbis test viruses

The pE2R1-GFP/2A Sindbis virus infectious clone (here referred to as SINV-GFP) expressing green fluorescent protein in the cytoplasm [35] was a kind gift from Dr. Hans Heidner. This infectious clone was used to generate a luciferase-expressing virus (SINV-Luc) by replacing GFP with luciferase using overlap PCR. Virus stocks were generated by in vitro RNA transcription and electroporation of BHK-21 cells as previously described [36], and titers were determined on BHK-21 cells.

### Initial multi-cycle RNAi screen

Screening was performed at the RNAi core at New York University Langone Medical Center using an arrayed library targeting 21,687 human genes in pools of 3 siRNAs per gene (Ambion Silencer® human genome siRNA library V3). RNAi pools were pre-arrayed in duplicate on Corning white polystyrene 384 well plates, and used to reverse-transfect U-2 OS cells (3000 cells/well) at a final concentration of 37.5 nM siRNA, 0.375% Lipofectamine RNAiMAX (Invitrogen) in medium without antibiotics. Six wells of each of the following control siRNAs were included on each plate: a nontargeting (NT) siRNA (Ambion Silencer® negative control #7), an siRNA targeting the clathrin heavy chain (CLTC) (Dharmacon clathrin heavy chain oligo I) [37], and an siRNA targeting RPL27A (Ambion siRNA ID#: 9257), which induces cell death by targeting a protein required for ribosome biogenesis [38], and which served as a control for transfection efficiency. Transfected cells were incubated for 48 h at 37°C, infected with SINV-Luc at a multiplicity of infection (MOI) of 1, and incubated for 24 h at 37°C, conditions optimized for multiple rounds of infection and the linear range of the assay (Figs. S1A and B). The SteadyGlo Luciferase Assay System reagent (Promega) was added to a final concentration of 25% and plates were read by an EnVision Multilabel plate reader (Perkin Elmer) using the luminescence aperture.

### Data analysis

Wells of interest from the initial screen were selected by analysis of the robust z-score and of the controls on each plate. Using CellHTS2 (software package implemented in Bioconductor/R), the raw data were first transformed in log-2 space and normalized to the per-plate median to remove systematic plate-to-plate variation. The robust z-score of each well was then calculated based on the median and median absolute deviation [39]. We also compared the experimental wells to the NT and clathrin siRNA controls on each plate, selecting those genes with lower luciferase activity than clathrin siRNA in both replicates, thereby reducing the likelihood of false positives. The ranked list resulting from these two criteria was manually curated to remove genes in which knockdown was previously reported to cause cell death, or in which luciferase levels were at background, indicative of cell death. We also eliminated genes that appeared to indirectly affect virus infection (e.g., ribosomal proteins). A final list of 400 genes in which infection was reduced by siRNA (corresponding to robust z-scores<−3) and 59 genes in which infection was promoted by siRNA (corresponding to robust z-scores>2) was selected for follow-up screening.

Microsoft EXCEL was used to calculate statistical significance by two-tailed unpaired Student's t-test.

### Follow-up screening and confirmation

siRNAs targeting the identified 459 genes were further tested individually for their effects on multi-cycle virus infection, and tested as pools for effects on cell viability and single cycle infection. Selected genes were also targeted by additional siRNAs or shRNAs, tested in HeLa and primary human endothelial cells, and evaluated for effects on other alphaviruses including SFV and CHIKV, and on VSV and DENV. These methods are described in detail in the supplementary information.

### Assays of steps in the alphavirus lifecycle

#### Assay for low pH-dependent fusion at the plasma membrane

U-2 OS cells were transfected as above with the indicated siRNAs in 384 well plates, and cultured for 48 h. Virus was pre-bound to cells on ice for 90 min and then pulsed with pH 5.5 medium for 1 min at 37°C to trigger fusion of virus with the cell plasma membrane [40]. Cells were cultured overnight at 28°C in the presence of 20 mM NH_4_Cl to prevent secondary infection, and infected cells scored by GFP expression or immunofluorescence. Fluorescence images were captured on a Zeiss Axiovert 200M microscope at 5X magnification. Infected cells were quantitated using Cell Profiler v. 2.0 r11710 cell image analysis software (Broad Institute) [41].

#### Viral RNA transfection

U-2 OS cells were transfected as above with the indicated siRNAs in 384 well plates, and cultured for 48 h. To test effects on viral RNA transcription/translation, cells were then transfected with SINV-mCherry RNA (a kind gift from Dr. G. Martinez in our lab) or SFV RNA using lipofectamine 2000 (Invitrogen) according to the manufacturer's instructions. Cells were incubated at 28°C for 16 h in the presence of 20 mM NH_4_Cl and infected cells were quantitated by fluorescence microscopy as described above.

#### Virus binding

U-2 OS cells seeded at 4×10^4^ cells/well on 8 well Lab-Tek chambered cover glass (Thermo Scientific) were transfected with 22.5 nM siRNA and 0.15% RNAiMax, and cultured for 48 h. To quantitate virus-cell surface binding, U-2 OS cells were washed 2X with Rmed (RPMI without bicarbonate, plus 10 mM HEPES and 0.2% BSA, pH 6.8). SINV-Luc (MOI = 10) or purified SFV (0.25 µg/well) in Rmed were pre-bound to U-2 OS cells on ice. Cells were then washed once with Rmed, fixed with 3% paraformaldehyde (PFA) in PBS, and bound virus was detected by staining with anti-SINV mAbs R2 and R6 or anti-SFV E1/E2 pAb followed by goat anti-mouse or goat anti-rabbit Ig Alexa fluor 488, respectively. Z-stack images were captured using a Leica SP2 confocal microscope, and brightfield images were used to determine the cell boundaries. Bound virus was quantified using Image J to determine the area of the virus particles bound per 200 cells, and normalized to the value obtained with the NT control. Commercially available antibodies against NRAMP2 were not able to detect cell surface expression of NRAMP2 in U-2 OS or HeLa cells (data not shown), precluding direct tests of receptor levels.

#### Virus endocytosis

U-2 OS cells were transfected as described for the virus binding assay. Purified SFV (0.25 µg/well) in Rmed pH 6.8 was bound to U-2 OS cells for 90 minutes on ice. The cells were incubated at 37°C in Rmed pH 7.4 for 30 min to permit endocytosis, washed on ice 3X with PBS and fixed with 3% PFA. Cell surface virus was stained with anti-SFV E1/E2 pAb and goat anti-rabbit Ig Alexa fluor 568. Cells were then permeabilized with 0.1% Triton X-100 and intracellular virus was stained with anti-SFV E1/E2 rabbit pAb and goat anti-rabbit Alexa fluor 488. Fluorescence microscopy was performed as described for the binding assay. Endocytosed particles containing only green stain were counted manually in 200 cells, and data normalized to the value obtained for the NT control.

#### Assay for conversion of E1 to the acid-induced conformation

U-2 OS cells were transfected as described for the virus binding assay. Purified SFV (0.25 µg/well) in Rmed pH 6.8 was bound to cells for 90 min on ice. The cells were then incubated at 37°C for 20 min to permit endocytosis of bound virus, washed 3X with ice-cold PBS and fixed with 3% PFA. Cells were permeabilized with 0.1% Triton X-100 and stained with mAb E1a-1, a previously described monoclonal antibody to the acid conformation of SFV E1 [25], followed by anti-mouse Ig Alexa fluor 568. Virus particles were detected and quantified as described for the binding assay, counting 200 cells/condition.

#### Lipid mixing virus fusion assay

Purified SFV (25 µg) was labeled by incubating with a final concentration of 7.5 µM 1,1′-Dioctadecyl-3,3,3′,3′-Tetramethylindodicarbocyanine, 4-Chlorobenzenesulfonate (DiD) in 50 µl HNE buffer (5 mM HEPES, 150 mM NaCl, 0.1 mM EDTA, pH 7.0) for 10 min at RT with intermittant vortexing. DiD incorporation was essentially complete at this concentration of dye, and no difference in the results was observed when labeled virus was passed over a desalting column. Labeled virus was therefore used without further purification. U-2 OS cells were incubated for 90 min on ice with 1 µg/well of DiD-labeled SFV in Rmed pH 6.8. The medium was changed to Rmed pH 7.4 and the samples warmed to 37°C for 20 min to permit endocytic uptake of bound virus. Cells were washed with PBS, fixed with 3% PFA, and evaluated for dequenching of DiD as described [42] for the bulk fusion assay. In brief, Z-stacks of 10 fields of cells per experiment were imaged using the 633 nm helium-neon laser on a Leica SP2 or SP5 confocal microscope, and the total fluorescent signal per imaging field was quantitated using Image J software, and normalized for cell number. From 400–600 cells were quantitated per condition. Cells were then permeabilized and stained with mAb E1a-1 as above to detect the low pH conformation of E1. The level of DiD staining was corrected by using mAb E1a-1 staining to adjust to equivalent levels of internalized, acid-triggered virus in each sample. Fusion values were then compared to those obtained for the NT control. Negligible fusion was detected in parallel NT samples treated with 20 mM NH_4_Cl to inhibit endosomal acidification (data not shown).

### Assays of the cellular endocytic pathway

U-2 OS cells were transfected on 8 well chambered cover glass as described for the virus binding assay and then treated as follows:

#### Transferrin uptake

Cells were prebound on ice for 1 h with 20 µg/ml Alexa fluor 568-conjugated human transferrin in McCoy's 5A medium without serum, incubated for 30 min at 37°C, washed once with 0.5M NaCl, 0.2M acetic acid, pH 2.5, then 2X with PBS, and fixed with 3% PFA. Internalized transferrin, LDL, and dextran (described below) were quantitated with Image J as described for the virus binding assay.

#### LDL uptake

Cells were incubated in McCoy's 5A medium with 0.1% BSA without serum overnight to induce LDL-receptor expression, and then were prebound on ice for 1 h with 10 µg/ml of DiI-labeled human LDL in McCoy's 5A in the absence of serum. The cells were then incubated for 1 h at 37°C, washed 2X with 4 mg/ml dextran sulfate pH 6.5, then 1x with PBS, and fixed with 3% PFA.

#### Dextran uptake

Cells were incubated with 0.5 mg/ml Alexa fluor 568-conjugated dextran (10,000 MW) in U-2 OS medium for 3 h at 37°C, washed, cultured in dextran-free U-2 OS medium for 2 h, washed 3X with PBS and fixed with 3% PFA.

#### Lysotracker assay

U-2 OS cells on 384 wells plates were transfected with siRNAs as described previously. Cells were incubated with 100 nM lysotracker red DND-99 in U-2 OS medium for 1 h at 37°C, transferred to U-2 OS medium without lysotracker, and imaged immediately at 20x magnification using the Zeiss Axiovert 200M microscope. All probes were from Invitrogen/Life Technologies.

## Supporting Information

Figure S1 **Optimization of screen parameters and distribution of screen data.** A. Optimization of multi-cycle SINV-Luc infection of U-2 OS cells. U-2 OS cells were cultured on a 384-well plate for 48 h, then infected with SINV-Luc at an MOI = 1. To prevent secondary infection, 20 mM NH_4_Cl was added to one set of wells at 2 h post-infection. Luciferase expression was quantitated at 24 h post-infection. In the absence of NH_4_Cl, the signal reflects both primary and secondary infection. Results shown are the average of eight samples +/− SEM. B. Optimization of control siRNA transfection. U-2 OS cells were transfected with siRNAs targeting the clathrin heavy chain (clathrin), ribosomal protein L27A (RPL27A), or a non-targeting control siRNA (NT). At 48 h post-transfection, cells were infected with SINV-Luc (MOI = 1). Luciferase expression was scored at 24 h-post-infection. Results shown are the average of eight samples +/− SEM. C. Distribution of screen data. The density distribution of the screen was generated by CellHTS2 as described in the methods. In brief, raw values were log2 transformed and plotted by robust z-score, based on plate median and median absolute deviation. D. Correlation between replicate plates in the screen. Robust z-scores (as in S3A) of representative duplicate plates were plotted. The Spearman rank correlation (SRC) for these replicate plates and the average SRC for the complete screen were calculated. E. Optimization of single-cycle SINV-Luc infection of U-2 OS cells. U-2 OS cells were transfected with the indicated siRNAs. At 72 h post transfection, cells were infected with SINV-Luc at an MOI = 10. 20 mM NH_4_Cl was added at 3 h post-infection to prevent secondary infection. Luciferase expression was scored at 9 h post-infection. Results shown are the average of eight samples +/− SEM. The comparable signal +/− NH_4_Cl confirms that assay is primarily scoring single-cycle infection.
(TIF)

Figure S2 **Effects of esiRNA and shRNA on virus infection.** U-2 OS cells were transfected with ARCN1 or RLUC control esiRNA for 48 h (A, D) or transduced with FUZ or TSPAN9 shRNA vectors for 14 days (B, C, E). mRNA levels of ARCN1, FUZ, or TSPAN9 were determined by Quantigene assay (A, B, C, respectively), performed in duplicate. SINV-GFP infection (MOI = 1, 24 h) was quantitated by GFP fluorescence and microscopy (D, E), and normalized to the indicated controls. D and E represent the mean +/− SEM of three experiments. (*p<0.05, **p<0.01, ***p<0.001).
(TIF)

Figure S3 **Effect of ARCN1 depletion on virus-cell binding and RNA-mediated infection.** A. The effect of ARCN1, FUZ, and TSPAN9 depletion on SFV binding. U-2 OS cells were transfected with the indicated siRNAs, and incubated for 48 h. SFV was bound to cells on ice and detected by immunofluorescence. Confocal extended focus images are shown with cell borders marked (bar = 10 µM). B, C. Effect of ARCN1 depletion on infection by transfected viral RNA. U-2 OS cells were transfected with the indicated siRNAs, incubated for 48 h, and transfected with SINV-mcherry (B) or SFV (C) viral RNA. Cells were incubated in the presence of 20 mM NH4Cl to block secondary virus infection. Infected cells were quantitated by fluorescence microscopy. Bar graph represents the mean +/− SEM of 3 experiments with data normalized to NT control (*p<0.05, **p<0.01).
(TIF)

Figure S4 **LDL uptake.** U-2 OS cells were transfected as in Fig. 2 A. Cells were pre-bound with fluorescent LDL on ice, incubated for 1 h at 37°C to permit endocytosis, and washed with dextran sulfate to remove non-internalized LDL before fixation and quantitation. The dextran sulfate wash sample was stripped with dextran sulfate prior to 37°C incubation. (*p<0.05, ***p<0.001). Bar = 10 µM.
(TIF)

Figure S5 **Localization and overexpression of TSPAN9.** A. Localization of TSPAN9. Clonal U-2 OS cells stably transfected with a control (U-2 OS-pcDNA) or TSPAN9 (U-2 OS-TSPAN9) expression vector were stained with anti-TSPAN9 pAb and nuclei were stained with Hoechst. Both panels show a single confocal slice from the center of the cell (bar = 10 µM). B. Effect of TSPAN9 overexpression on SINV infection. U-2 OS-pcDNA or U-2 OS-TSPAN9 cells were infected with SINV-GFP virus. Infection was quantitated by fluorescence microscopy at 24 h postinfection. Data shown are the mean and SE of 4 independent experiments, with infection normalized to that of the control cells. Infection was increased by 2–6 fold over control in each experiment.
(TIF)

Table S1 **Primary RNAi screen dataset for SINV.**
(XLSX)

Table S2 **Human genes identified by the screen as promoting SINV-Luc infection.**
(XLSX)

Table S3 **Human genes identified by the screen as inhibiting SINV-Luc infection.**
(XLSX)

Table S4 **Comparison of human genes involved in SINV-Luc infection and endocytic pathway genes.**
(DOCX)

Table S5 **Comparison of human genes involved in SINV-Luc infection versus infection by other viruses.**
(DOCX)

Text S1 **Supplementary methods.**
(DOCX)
