# Supplementary figures and images for: Genome-Wide RNAi Screen Identifies Novel Host Proteins Required for Alphavirus Entry
Source: PLoS Pathog. 2013 Dec 19;9(12):e1003835. doi: 10.1371/journal.ppat.1003835 (PMC3868536; doi:10.1371/journal.ppat.1003835)

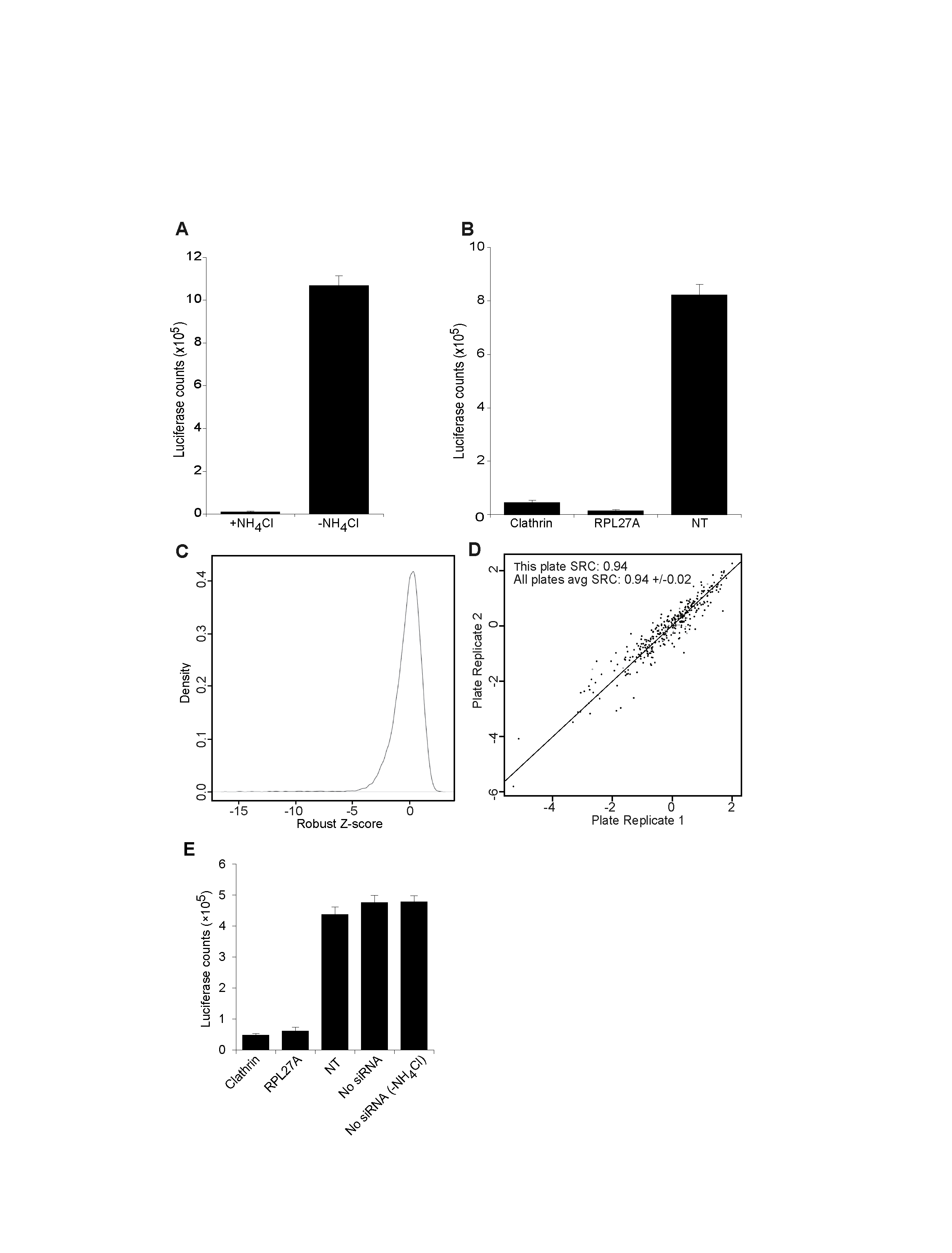

Supplement: Figure S1 — Optimization of screen parameters and distribution of screen data. A. Optimization of multi-cycle SINV-Luc infection of U-2 OS cells. U-2 OS cells were cultured on a 384-well plate for 48 h, then infected with SINV-Luc at an MOI = 1. To prevent secondary infection, 20 mM NH4Cl was added to one set of wells at 2 h post-infection. Luciferase expression was quantitated at 24 h post-infection. In the absence of NH4Cl, the signal reflects both primary and secondary infection. Results shown are the average of eight samples +/− SEM. B. Optimization of control siRNA transfection. U-2 OS cells were transfected with siRNAs targeting the clathrin heavy chain (clathrin), ribosomal protein L27A (RPL27A), or a non-targeting control siRNA (NT). At 48 h post-transfection, cells were infected with SINV-Luc (MOI = 1). Luciferase expression was scored at 24 h-post-infection. Results shown are the average of eight samples +/− SEM. C. Distribution of screen data. The density distribution of the screen was generated by CellHTS2 as described in the methods. In brief, raw values were log2 transformed and plotted by robust z-score, based on plate median and median absolute deviation. D. Correlation between replicate plates in the screen. Robust z-scores (as in S3A) of representative duplicate plates were plotted. The Spearman rank correlation (SRC) for these replicate plates and the average SRC for the complete screen were calculated. E. Optimization of single-cycle SINV-Luc infection of U-2 OS cells. U-2 OS cells were transfected with the indicated siRNAs. At 72 h post transfection, cells were infected with SINV-Luc at an MOI = 10. 20 mM NH4Cl was added at 3 h post-infection to prevent secondary infection. Luciferase expression was scored at 9 h post-infection. Results shown are the average of eight samples +/− SEM. The comparable signal +/− NH4Cl confirms that assay is primarily scoring single-cycle infection. (TIF) [file ppat.1003835.s001.tif]

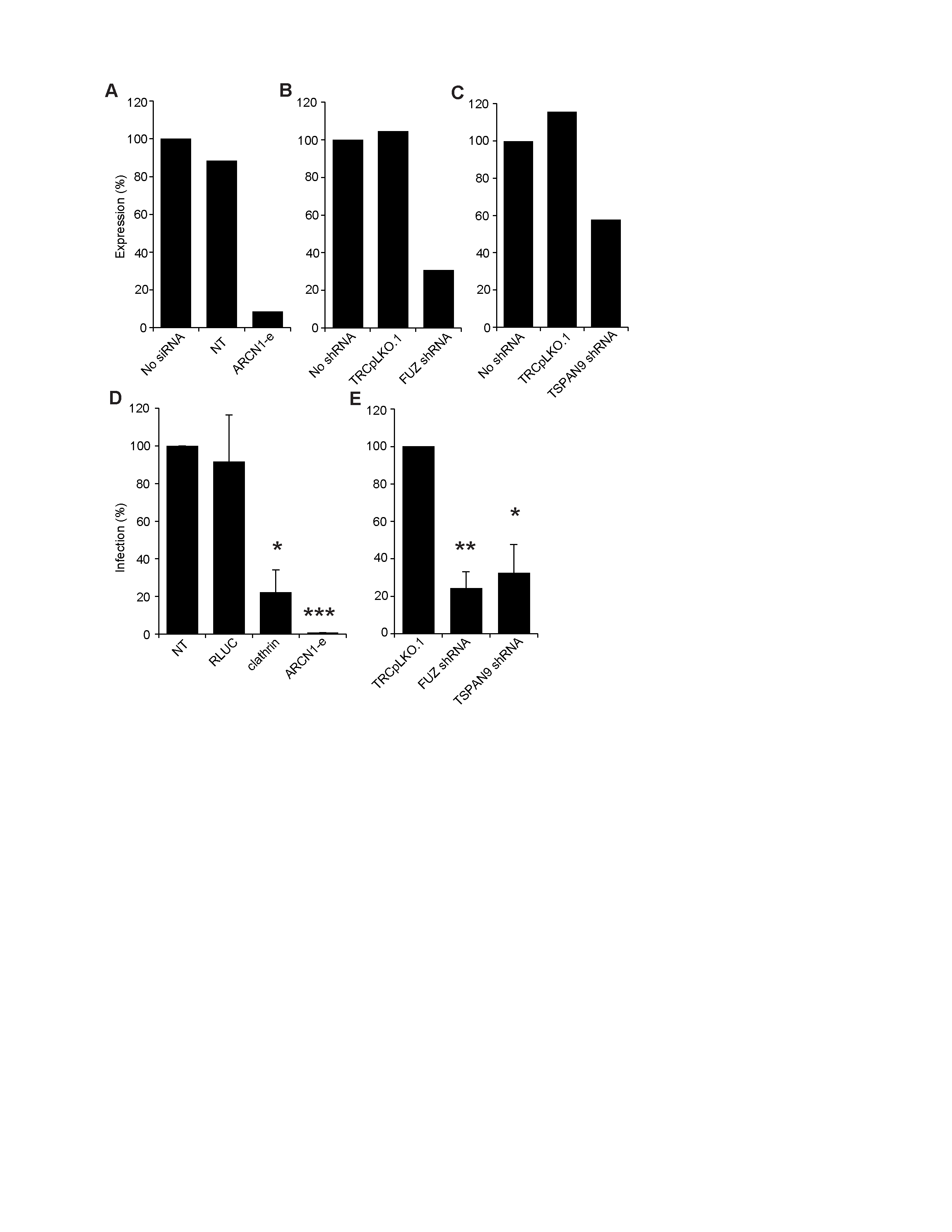

Supplement: Figure S2 — Effects of esiRNA and shRNA on virus infection. U-2 OS cells were transfected with ARCN1 or RLUC control esiRNA for 48 h (A, D) or transduced with FUZ or TSPAN9 shRNA vectors for 14 days (B, C, E). mRNA levels of ARCN1, FUZ, or TSPAN9 were determined by Quantigene assay (A, B, C, respectively), performed in duplicate. SINV-GFP infection (MOI = 1, 24 h) was quantitated by GFP fluorescence and microscopy (D, E), and normalized to the indicated controls. D and E represent the mean +/− SEM of three experiments. (*p<0.05, **p<0.01, ***p<0.001). (TIF) [file ppat.1003835.s002.tif]

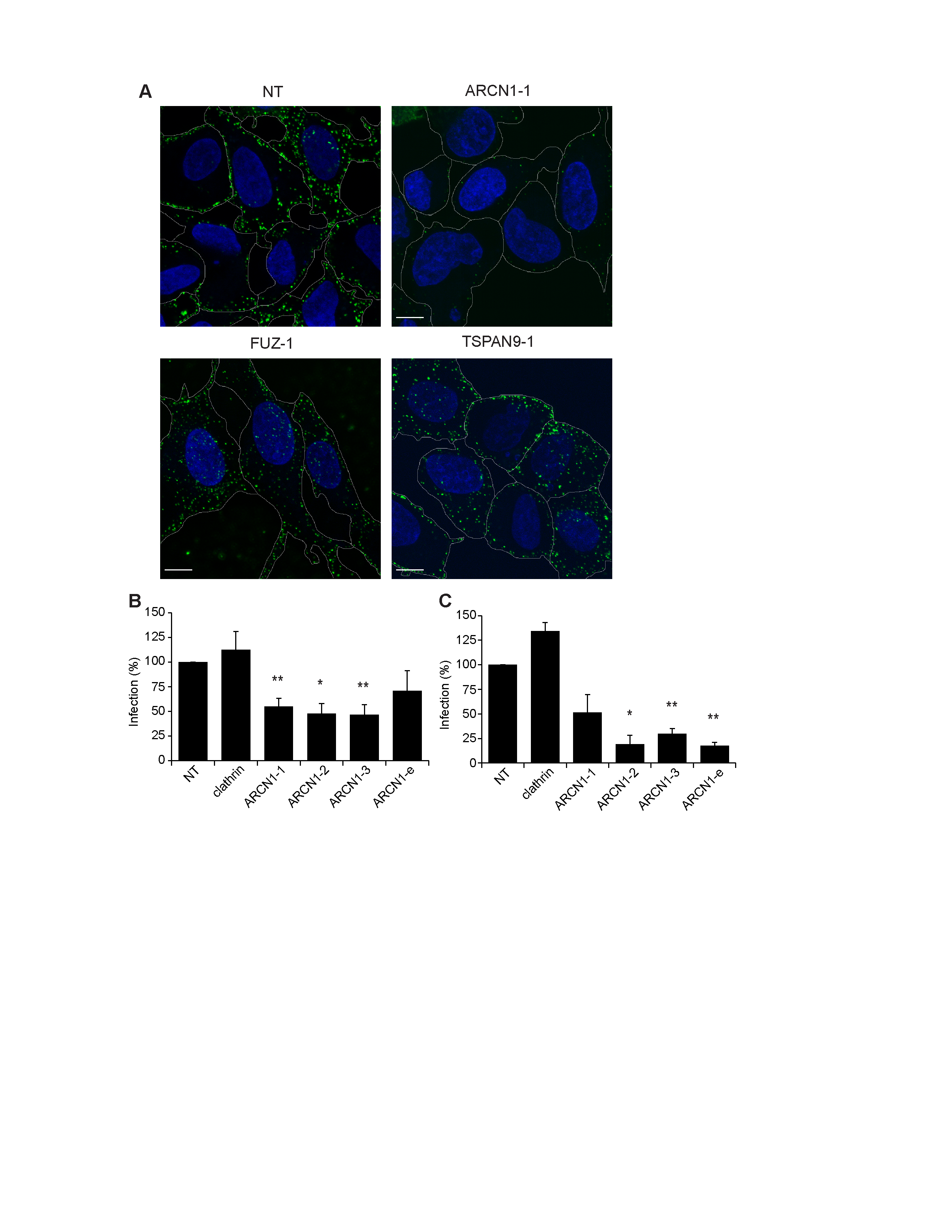

Supplement: Figure S3 — Effect of ARCN1 depletion on virus-cell binding and RNA-mediated infection. A. The effect of ARCN1, FUZ, and TSPAN9 depletion on SFV binding. U-2 OS cells were transfected with the indicated siRNAs, and incubated for 48 h. SFV was bound to cells on ice and detected by immunofluorescence. Confocal extended focus images are shown with cell borders marked (bar = 10 µM). B, C. Effect of ARCN1 depletion on infection by transfected viral RNA. U-2 OS cells were transfected with the indicated siRNAs, incubated for 48 h, and transfected with SINV-mcherry (B) or SFV (C) viral RNA. Cells were incubated in the presence of 20 mM NH4Cl to block secondary virus infection. Infected cells were quantitated by fluorescence microscopy. Bar graph represents the mean +/− SEM of 3 experiments with data normalized to NT control (*p<0.05, **p<0.01). (TIF) [file ppat.1003835.s003.tif]

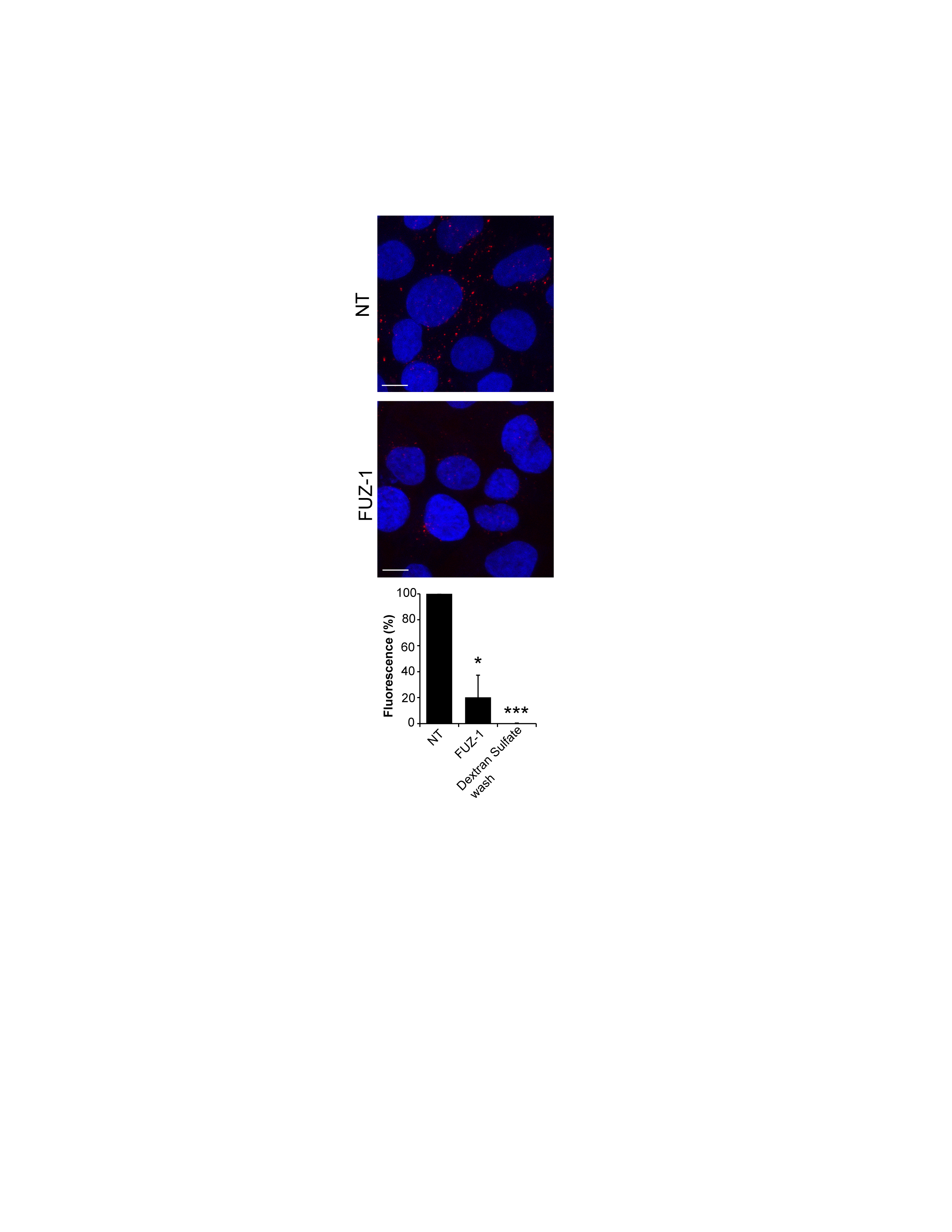

Supplement: Figure S4 — LDL uptake. U-2 OS cells were transfected as in Fig. 2 A. Cells were pre-bound with fluorescent LDL on ice, incubated for 1 h at 37°C to permit endocytosis, and washed with dextran sulfate to remove non-internalized LDL before fixation and quantitation. The dextran sulfate wash sample was stripped with dextran sulfate prior to 37°C incubation. (*p<0.05, ***p<0.001). Bar = 10 µM. (TIF) [file ppat.1003835.s004.tif]

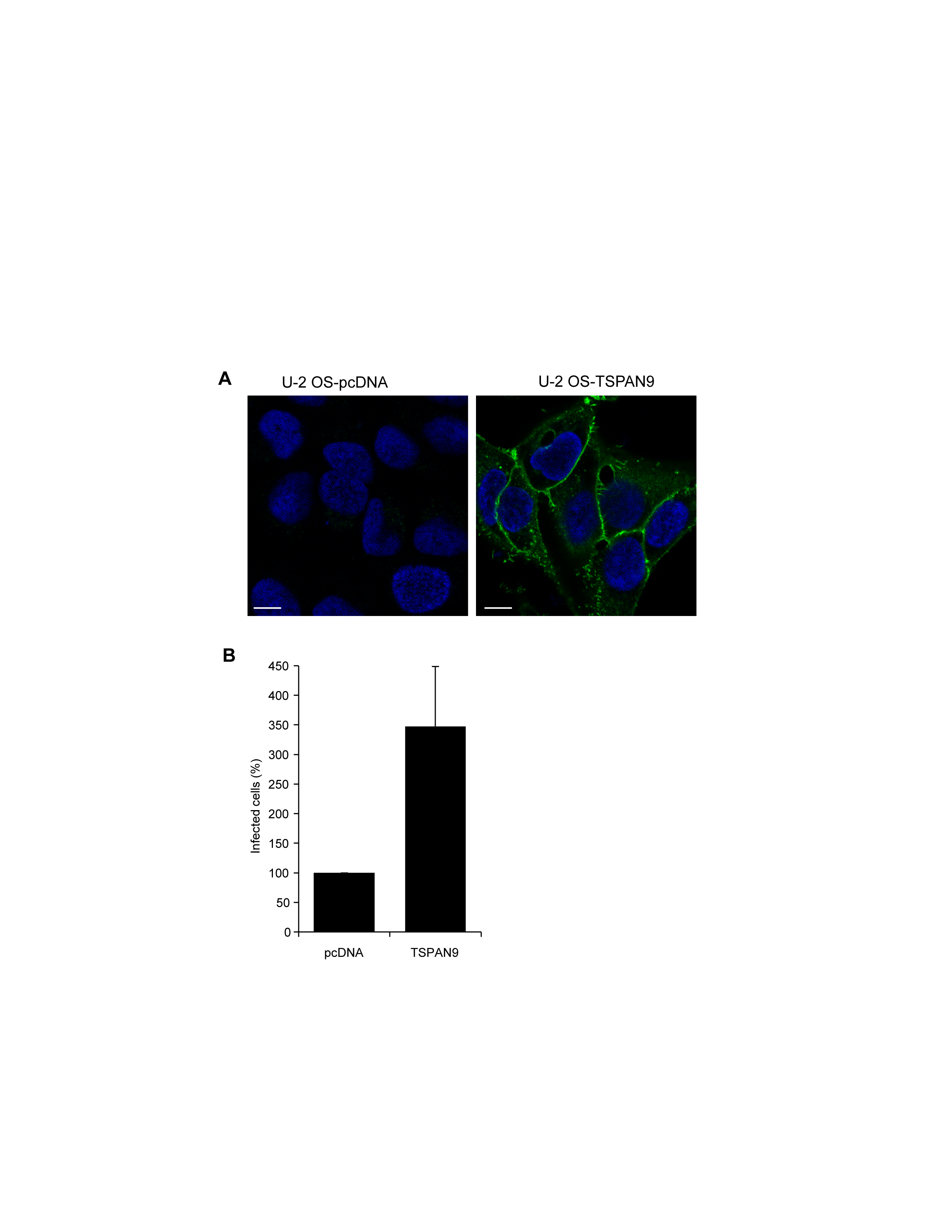

Supplement: Figure S5 — Localization and overexpression of TSPAN9. A. Localization of TSPAN9. Clonal U-2 OS cells stably transfected with a control (U-2 OS-pcDNA) or TSPAN9 (U-2 OS-TSPAN9) expression vector were stained with anti-TSPAN9 pAb and nuclei were stained with Hoechst. Both panels show a single confocal slice from the center of the cell (bar = 10 µM). B. Effect of TSPAN9 overexpression on SINV infection. U-2 OS-pcDNA or U-2 OS-TSPAN9 cells were infected with SINV-GFP virus. Infection was quantitated by fluorescence microscopy at 24 h postinfection. Data shown are the mean and SE of 4 independent experiments, with infection normalized to that of the control cells. Infection was increased by 2–6 fold over control in each experiment. (TIF) [file ppat.1003835.s005.tif]
