# Supplementary material for: Genome-Wide RNAi Screen Identifies Novel Host Proteins Required for Alphavirus Entry
Source: PLoS Pathog. 2013 Dec 19;9(12):e1003835. doi: 10.1371/journal.ppat.1003835 (PMC3868536; doi:10.1371/journal.ppat.1003835)
Supplement: Table S4 — Comparison of human genes involved in SINV-Luc infection and endocytic pathway genes. (DOCX) [file ppat.1003835.s009.docx]

| **Table S4. Comparison of human genes involved in SINV-Luc infection and endocytic pathway genes.** | | | | | |
| --- | --- | --- | --- | --- | --- |
| Confirmed genes shared by SINV infection & endocytosis screens (total of 44 genes) | | | | | |
| Genes shared by SINV & both endocytosis screens (4 genes) | | Genes shared by SINV & clathrin-coated vesicle screens [1] (15 genes) | | Genes shared by SINV & endocytosis screens [2] (25 genes) | |
| AGTRAP |  | ADRM1 |  | ACAP1 | MRE11A |
| DNM2*^/^** |  | ALPP |  | BCL2L1 | NDST1 |
| ITFG3 |  | ARCN1 |  | C11orf68 | NEK2 |
| YARS |  | ATP6V0C |  | CHMP2B | OR6N2 |
|  |  | CTDSP1 |  | CKS2 | PLAT |
|  |  | GNAI3 |  | CSF2 | PPP1R12B |
|  |  | ITSN1* |  | FBXL5 | PRM1 |
|  |  | LIMA1 |  | FLT4 | TAF5 |
|  |  | MAP2K3 |  | FOS | TAF4 |
|  |  | NPEPL1 |  | FURIN | TSPAN9 |
|  |  | OGDH |  | GCNT3 |  |
|  |  | PAFAH1B2 |  | GPC3 |  |
|  |  | SRRT |  | GPR52 |  |
|  |  | STK10 |  | INTS7 |  |
|  |  | TOMM34 |  | LZTR1 |  |
|  |  |  |  |  |  |
|  |  |  |  |  |  |
| * Predicted CCV proteins | | | | | |
| ** Clathrin cage associated proteins | | | | | |

Overlap between the validated genes listed in Tables S2 and S3 and those identified as being involved in clathrin-coated vesicles (middle column) [1] or in the endocytic uptake pathways of transferrin and epidermal growth factor (right column) [2], or in both (left column).

1. Borner GH, Antrobus R, Hirst J, Bhumbra GS, Kozik P, et al. (2012) Multivariate proteomic profiling identifies novel accessory proteins of coated vesicles. J Cell Biol 197: 141-160.

2. Collinet C, Stoter M, Bradshaw CR, Samusik N, Rink JC, et al. (2010) Systems survey of endocytosis by multiparametric image analysis. Nature 464: 243-249.
