# Supplementary material for: Genome-Wide RNAi Screen Identifies Novel Host Proteins Required for Alphavirus Entry
Source: PLoS Pathog. 2013 Dec 19;9(12):e1003835. doi: 10.1371/journal.ppat.1003835 (PMC3868536; doi:10.1371/journal.ppat.1003835)
Supplement: Table S5 — Comparison of human genes involved in SINV-Luc infection versus infection by other viruses. (DOCX) [file ppat.1003835.s010.docx]

| **Table S5. Comparison of human genes involved in SINV-Luc infection versus infection by other viruses.** | | | | |
| --- | --- | --- | --- | --- |
| **Gene** | **SINV Host Factor** | **Other Virus** | **Antiviral / Pro-viral** | **References** |
| AGTRAP | Pro-viral | Influenza Virus | Pro-viral | [1] |
| ARCN1 | Pro-viral | HCV | Pro-viral | [2] |
|  |  | Influenza Virus | Pro-viral | [3,4] |
|  |  | Rotavirus | Pro-viral | [5] |
|  |  | VSV | Pro-viral | [6] |
| ATP6V0C | Pro-viral | DENV | Pro-viral | [7] |
|  |  | Influenza Virus | Pro-viral | [4,8] |
|  |  | Rotavirus | Pro-viral | [5] |
|  |  | WNV | Pro-viral | [9] |
| BCL2L1 | Pro-viral | Coxsackievirus B | Pro-viral | [10] |
| CHMP2B | Pro-viral | Influenza Virus | Pro-viral | [3] |
| CSF2 | Pro-viral | Vaccinia Virus | Antiviral | [1] |
| CTDSP1 | Antiviral | Rotavirus | Pro-viral | [5] |
| DBNDD1 | Pro-viral | HCV | Pro-viral | [11] |
| DNM2 | Pro-viral | Coxsackievirus B | Pro-viral | [10] |
|  |  | Poliovirus | Pro-viral | [10] |
| FBXL5 | Antiviral | Rotavirus | Pro-viral | [5] |
| FLT4 | Pro-viral | HCV | Pro-viral | [11] |
|  |  | Influenza Virus | Pro-viral | [4] |
| FNTA | Antiviral | HIV | Pro-viral | [12] |
| INTS7 | Pro-viral | HIV | Pro-viral | [12] |
| ITFG3 | Antiviral | WNV | Pro-viral | [9] |
| MAP2K3 | Antiviral | Influenza Virus | Pro-viral | [4] |
| MICB | Pro-viral | HIV | Antiviral & Pro-viral | [13] |
| MRE11A | Antiviral | HIV | Antiviral & Pro-viral | [13] |
| NDST1 | Pro-viral | WNV | Pro-viral | [9] |
|  |  | Vaccinia Virus | Pro-viral | [1] |
| NOS3 | Antiviral | Influenza Virus | Pro-viral | [3] |
| PPP1R12B | Antiviral | Rotavirus | Pro-viral | [5] |
| SH3BP5L | Pro-viral | Rotavirus | Pro-viral | [5] |
| TAF4 | Antiviral | Vaccinia Virus | Antiviral | [1] |
| TAF5 | Antiviral | Vaccinia Virus | Antiviral & Pro-viral | [1] |
| TSPAN9 | Pro-viral | Vaccinia Virus | Antiviral | [1] |

Overlap between the validated genes listed in Tables S2 and S3 and those identified by screens for genes involved in infection by the indicated viruses.

1. Sivan G, Martin SE, Myers TG, Buehler E, Szymczyk KH, et al. (2013) Human genome-wide RNAi screen reveals a role for nuclear pore proteins in poxvirus morphogenesis. ProcNatlAcadSciUSA 110: 3519-3524.

2. Tai AW, Benita Y, Peng LF, Kim SS, Sakamoto N, et al. (2009) A functional genomic screen identifies cellular cofactors of hepatitis C virus replication. Cell Host Microbe 5: 298-307.

3. Brass AL, Huang IC, Benita Y, John SP, Krishnan MN, et al. (2009) The IFITM proteins mediate cellular resistance to influenza A H1N1 virus, West Nile virus, and dengue virus. Cell 139: 1243-1254.

4. Konig R, Stertz S, Zhou Y, Inoue A, Hoffmann HH, et al. (2010) Human host factors required for influenza virus replication. Nature 463: 813-817.

5. Silva-Ayala D, Lopez T, Gutierrez M, Perrimon N, Lopez S, et al. (2013) Genome-wide RNAi screen reveals a role for the ESCRT complex in rotavirus cell entry. ProcNatlAcadSciUSA 110: 10270-10275.

6. Panda D, Das A, Dinh PX, Subramaniam S, Nayak D, et al. (2011) RNAi screening reveals requirement for host cell secretory pathway in infection by diverse families of negative-strand RNA viruses. ProcNatlAcadSciUSA 108: 19036-19041.

7. Sessions OM, Barrows NJ, Souza-Neto JA, Robinson TJ, Hershey CL, et al. (2009) Discovery of insect and human dengue virus host factors. Nature 458: 1047-1050.

8. Karlas A, Machuy N, Shin Y, Pleissner KP, Artarini A, et al. (2010) Genome-wide RNAi screen identifies human host factors crucial for influenza virus replication. Nature 463: 818-822.

9. Krishnan MN, Ng A, Sukumaran B, Gilfoy FD, Uchil PD, et al. (2008) RNA interference screen for human genes associated with West Nile virus infection. Nature 455: 242-245.

10. Coyne CB, Bozym R, Morosky SA, Hanna SL, Mukherjee A, et al. (2011) Comparative RNAi screening reveals host factors involved in enterovirus infection of polarized endothelial monolayers. Cell Host Microbe 9: 70-82.

11. Li Q, Brass AL, Ng A, Hu Z, Xavier RJ, et al. (2009) A genome-wide genetic screen for host factors required for hepatitis C virus propagation. ProcNatlAcadSciUSA 106: 16410-16415.

12. Brass AL, Dykxhoorn DM, Benita Y, Yan N, Engelman A, et al. (2008) Identification of host proteins required for HIV infection through a functional genomic screen. Science 319: 921-926.

13. Zhou H, Xu M, Huang Q, Gates AT, Zhang XD, et al. (2008) Genome-scale RNAi screen for host factors required for HIV replication. Cell Host Microbe 4: 495-504.
