# Supplementary material for: Genome-Wide RNAi Screen Identifies Novel Host Proteins Required for Alphavirus Entry
Source: PLoS Pathog. 2013 Dec 19;9(12):e1003835. doi: 10.1371/journal.ppat.1003835 (PMC3868536; doi:10.1371/journal.ppat.1003835)
Supplement: Text S1 — Supplementary methods. (DOCX) [file ppat.1003835.s011.docx]

**Text S1.**

**Supplementary Methods:**

**Follow-up screens.** To confirm the results of the initial screen, the siRNAs from the original pool of 3 siRNAs were tested individually for their effects on virus infection. This screen was performed in triplicate on U-2 OS cells using the same procedure as the initial screen.

To eliminate genes whose knockdown caused cell death, U-2 OS cells were reverse transfected with pools of 3 siRNAs/gene in duplicate using the same protocol as in the initial screen. After transfection, cells were incubated for 72 h at 37°C to produce maximal effects of siRNA knockdown on cell viability, as determined by optimization with the RPL27A siRNA control. CellTiterGlo (Promega) was added to a final concentration of 31% and plates were read using the EnVision reader. The values were normalized using the positive cell death control, RPL27A (Ambion siRNA ID#: 9257), and the negative NT control on each plate. Normalized values were then used in the following formula: (positive control – xi)/(positive control – negative control), where positive control = median of RPL27A signals, negative control = median of NT signals, and xi = signal in each well. Values less than 0.5 were considered to reflect significantly decreased cell numbers and these genes were excluded from further study.

A single cycle infection screen was performed to differentiate genes involved in the initial steps of infection (Fig. S3). U-2 OS cells were reverse transfected with pools of 3 siRNAs/gene in duplicate using the same protocol as in the initial screen. Cells were incubated for 72 h at 37°C, then were infected with SINV-Luc at MOI=10 for 3 h to infect the majority of cells. 20 mM NH_4_Cl was then added to the culture medium to prevent secondary infection. Cells were incubated for an additional 6 h and luciferase expression was quantitated as above. Out of 271 siRNA pools that decreased infection, 256 also showed reduced infection in the single cycle screen. Out of 59 genes in the original set with increased infection, 39 continued to show increased infection in the single cycle screen.

**siRNA and shRNA sequences.** The individual siRNAs from the Ambion library were designated as ARCN1-1, -2 or -3 (#1 – 10330, GGCUCUUCUCAAGAGUGAUtt; #2 - 10234, GGGCUUAUUAGCAGCUUUUtt; #3 – 10423, GGAAAUGGCACAAACAUAAtt), FUZ-1, -2, or -3 (#1 – 32375, GGACUUGAGGGCCAGUUAUtt; #2 – 125594, GCCAGUUGUAUCCACAGCUtt; #3 – 32471, GGGCCAGUUAUUGCCUCAUtt), and TSPAN9-1, -2, or -3 (#1 – 18435, GGCUUUUUAUACCUUACAAtt; #2 – 18253, GGCAACUUUGCCACCUUCUtt; #3 – 18345, GGAAAACAAGUGCCUCCUCtt). The siRNA sequences used for detailed follow up studies are underlined. ARCN1-e and RLUC indicate Mission esiRNAs targeting human ARCN1 or luciferase (Sigma), each a heterogeneous mixture of siRNAs targeting the mRNA sequence.

shRNA expression constructs targeting FUZ (TRCN0000128880, sequence GATAAAGAGCCTTCACCAGAA) and TSPAN9 (TRCN0000119247, sequence CCCAGTTATTTCACAGACATT) were selected from the TRC library (Open Biosystems). The TRCpLKO.1 vector served as a negative control.

**Lentivirus packaging.** The indicated shRNA expression plasmids were packaged into HIV-1-derived lentiviral particles pseudotyped with VSV G protein, using the third generation system plasmids pRSV-Rev, pMD2 VSVG, and pMDLg/pRRE. 2 h prior to transfection, 60% confluent 293T cells on a 10 cm dish were transferred to IMDM+GlutaMAX-1 medium (Gibco Life Technologies) containing 10% FBS, 100 U penicillin/ml, and 100 μg streptomycin/ml. Cells were then co-transfected with all four plasmids using calcium phosphate [43]. Supernatants containing the lentiviral particles were collected at 24 h and 48 h post-transfection, aliquoted, and stored at -80°C.

**Inhibition of gene expression using shRNA constructs.** shRNA expression constructs targeting FUZ and TSPAN9 (TRC library, Open Biosystems), or the TRCpLKO.1 vector alone were packaged into HIV-1-derived lentiviral particles pseudotyped with VSV G protein, as described in the Supplemental Information. U-2 OS cells were then transduced as follows: 1x10^5^ U-2 OS cells in a 6 well plate were treated with 0.5 ml U-2 OS cell medium containing 8 μg/ml polybrene plus 0.5 ml lentivirus supernatant. After 16 h, 1 ml U-2 OS medium was added to cells. Three days after the first transduction, a second transduction was done following the same protocol. Two sequential transductions were necessary for sufficient gene knockdown as determined by RNA quantitation and phenotype. The maximum phenotypic effect of gene knockdown was observed 10-11 days after the second transduction.

**RNA quantitation using branched DNA assay.** RNA levels in U-2 OS cells transfected with siRNA or transduced with shRNA were quantitated by the shRNA screening facility at Albert Einstein College of Medicine using the Quantigene Assay 2.0 (Affymetrix) with branched DNA probe sets and protocols as provided by the manufacturer (Panomics). RNA levels were measured in duplicate wells for each gene of interest and were normalized to the cellular gene PPIB.

**Infectivity tests of other viruses.** Tests of infection were performed on U-2 OS cells transfected as above with the indicated siRNAs in 384 well plates. Multiplicity, infection and detection conditions were optimized for each virus prior to assay, and are summarized as follows. Cells were infected with a plaque-purified SFV isolate [44] at 100 pfu/well (MOI=0.01) for 12 h, the CHIK virus vaccine strain 181/25 (kindly provided by Dr. Robert Tesh) at 1000 pfu/well (MOI=0.1) for 16 h, Dengue virus type 2 (DENV2) New Guinea C strain [45] at 5000 pfu/well (MOI=0.5) for 40 h, and VSV-GFP (kindly provided by Dr. Kartik Chandran) at 5000 pfu/well (MOI=0.5) for 16 h. The pE2S1-GFP/2A Sindbis virus infectious clone was kindly provided by Dr. Hans Heidner [46] and used to generate a virus stock (here referred to as SINV-pE2S1) and perform infection assays at 1000 pfu/well (MOI=0.1) for 24 h. All virus titers were determined by plaque assay or infectious center assay on BHK-21cells. SFV and CHIK-infected U-2 OS cells were detected by staining with an anti-SFV E1/E2 rabbit polyclonal antibody [44] and goat anti-rabbit Ig Alexa fluor 488. For DENV2, infected cells were stained with anti-DENV2 mouse hyperimmune serum (1:100) and goat anti-mouse Alexa fluor 488. Cells were also stained with Hoechst dye at 1:5000 in PBS. Fluorescence images were captured on a Zeiss Axiovert 200M microscope at 5X magnification. Quantitation of images was performed using Cell Profiler v. 2.0 r11710 cell image analysis software (Broad Institute) [47]. Results are shown in Fig. 3B for a single siRNA, but similar levels of inhibition were observed for all 3 siRNAs against ARCN1, TSPAN9 and FUZ.

**Infectivity tests on primary human cells.** Normal pooled human umbilical vein endothelial cells (HUVEC) were purchased from Lonza and maintained in endothelial cell growth media according to the company's recommendations. HUVECs (1500 cells per well; 384-well plate) were reverse-transfected with the indicated siRNAs at a final concentration of 37.5 nM, 0.15% Lipofectamine RNAiMAX (Invitrogen) in endothelial cell growth media. At 60 h post transfection, the cells were infected with either SFV at MOI of 0.5 or CHIKV at MOI of 5, incubated at 37°C for 8 h or 12 h, respectively, and infection quantitated by immunofluorescence as described above. These conditions monitored virus infection prior to significant secondary infection.

**Bioinformatics Analyses.** Official HUGO Gene Nomenclature Committee (HGNC) gene symbol, Entrez gene ID, Ensembl ID, and official HGNC full name were retrieved in batch from Genecards/GeneALaCart (www.genecards.org). Analyses of gene enrichment, networks, pathways, and biological functions were performed using Ingenuity Pathway Analysis (IPA).

**Generation of TSPAN9-overexpressing cell line.** TSPAN9 cDNA (Clone ID: HsCD00339824, Harvard DF/HCC DNA Resource Core plasmid repository - PlasmID) was cloned into pcDNA3.1(-) vector (Invitrogen) using the NheI and EcoRI restriction sites, and transfected into U-2 OS cells using Lipofectamine 2000 (Invitrogen). At 48 h post-transfection, geneticin (GIBCO) was added into the U-2 OS growth medium at the final concentration of 1 µg/ml to select for stable TSPAN9-overexpressing cells. A clonal cell line that homogenously overexpresses TSPAN9 was selected for subsequent study. As a control, a population of geneticin-resistant U-2 OS cells was generated by transfection with pcDNA3.1(-).

**Detection of TSPAN9 in U-2 OS cells.** TSPAN9-overexpressing U-2 OS line or the control U-2 OS cells generated using empty vector were fixed with 3% PFA and permeabilized with 0.1% Triton X-100. The cells were stained with pAb ab61261 (Abcam, Eugene, OR) followed by anti-rabbit Ig Alexa fluor 488, and Hoechst dye at 1:5000 in PBS.

**References**

43. Follenzi A, Naldini L (2002) Generation of HIV-1 derived lentiviral vectors. Meth Enzym 346: 454-465.

44. Vashishtha M, Phalen T, Marquardt MT, Ryu JS, Ng AC, et al. (1998) A single point mutation controls the cholesterol dependence of Semliki Forest virus entry and exit. J Cell Biol 140: 91-99.

45. Liao M, Kielian M (2005) Domain III from class II fusion proteins functions as a dominant-negative inhibitor of virus-membrane fusion. J Cell Biol 171: 111-120.

46. Thomas JM, Klimstra WB, Ryman KD, Heidner HW (2003) Sindbis virus vectors designed to express a foreign protein as a cleavable component of the viral structural polyprotein. J Virol 77: 5598-5606.

47. Kamentsky L, Jones TR, Fraser A, Bray MA, Logan DJ, et al. (2011) Improved structure, function and compatibility for CellProfiler: modular high-throughput image analysis software. Bioinformatics 27: 1179-1180.
